# Microsurgical clipping of ruptured wide-neck Aneurysms: A comparative analysis with Woven EndoBridge data

**DOI:** 10.1016/j.bas.2025.105631

**Published:** 2025-10-16

**Authors:** B. Kranawetter, T. Chacón-Quesada, D. Mielke, V. Malinova, V. Rohde, S. Hernández-Durán

**Affiliations:** a Department of Neurosurgery, University Medical Center, Georg-August University Göttingen, Germany; b Department of Neurosurgery, University Medical Center, University of Augsburg, Germany; c Department of Neurosurgery, Rostock University Medical Center, Germany

**Keywords:** Wide-neck bifurcation aneurysm, Woven EndoBridge device, Subarachnoid hemorrhage, Intracranial aneurysm, Microsurgical clipping

## Abstract

**Background:**

Due to their complex configuration, wide-neck aneurysms (WNAs) present significant challenges. The intrasaccular Woven EndoBridge (WEB) device was introduced in 2010. However, complete occlusion rates with WEB remain relatively low (40–60 %). The aim of this study was therefore to evaluate our institutional experience with microsurgical clipping of ruptured WNAs and to compare these results with a cohort of WNAs treated with the WEB device.

**Methods:**

A retrospective study of consecutive adult patients with ruptured WNAs who underwent microsurgical clipping at our institution between 2010 and 2020 was performed. Primary outcome measure was the complete occlusion of the aneurysm equivalent to a Raymond Roy Occlusion Classification (RROC) Class I. Subsequently, our institutional results were then compared with outcomes from the U.S. multicenter WEB study by Cortez et al.

**Results:**

87 patients with ruptured WNBAs were included. The most common aneurysm location was the anterior communicating artery (45/87, 52 %), followed by the middle cerebral artery bifurcation (38/87, 44 %), internal carotid artery terminus (2/87, 2 %), and basilar artery apex (2/87, 2 %). Mean follow-up in our cohort was 22.7 (±29.1) months. The procedure-related morbidity was low in both cohorts, 3.3 % (3/91) in the WEB cohort and 2.3 % (2/87) in our cohort (p = 1.00). However, the complete occlusion rate after microsurgical clipping was significantly higher (94 % (82/87)) compared to WEB (48 % (24/50) (p < 0.001)).

**Conclusions:**

The study demonstrates that microsurgical clipping is a safe and effective treatment method for ruptured WNAs aneurysms and that it is superior to WEB in terms of complete aneurysm occlusion.

## Highlights

• Microsurgical clipping achieves a 94 % complete occlusion rate in ruptured WNBAs.
• The WEB device shows significantly lower occlusion rates (48 %) compared to clipping.
• Both treatment methods have similar low procedure-related morbidity rates.
• This study is the first to directly compare clipping vs. WEB in ruptured WNBAs.
• Microsurgical clipping remains a superior option for complete aneurysm occlusion.

## Abbreviations

ACA: anterior cerebral artery
AComA: anterior communicating artery
CSF: cerebral spinal fluid
CTA: computed tomography angiography
EVD: external ventricular drain
ICG: indocyanide green
ICAt: internal carotid artery terminus
ICP: intracranial pressure
DSA: digital subtraction angiography
MCA: middle cerebral artery
MRA: magnetic resonance angiography
MRI: magnetic resonance imaging
PTA: percutaneous transluminal angioplasty
RROC: Raymond Roy Occlusion Classification
SSI: surgical site infections
TCD: transcranial Doppler
WOS: WEB occlusion scale
WNAs: wide-neck aneurysms
WEB: Woven EndoBridge
WNBAs: wide-neck bifurcation aneurysms

## 1 Introduction

Aneurysmal subarachnoid hemorrhage (SAH) is a life-threatening emergency associated with substantial mortality and morbidity (Hoh et al., 2023). The primary treatment objective is the definitive occlusion of the ruptured aneurysm, achieved either through microsurgical clipping or endovascular techniques (Hoh et al., 2023). Debate continues regarding the relative superiority of these approaches. Aneurysm morphology, anatomical location, and the patient's clinical status are the primary determinants in guiding treatment selection (Versyck et al., 2024). Despite these considerations, certain aneurysm types, such as wide-neck aneurysms (WNAs), remain especially challenging to manage with either strategy (Fiorella et al., 2017a; Pierot and Biondi, 2016). WNAs are commonly defined by a neck diameter of ≥4 mm and/or a dome-to-neck ratio of <2 (Hendricks et al., 2019; Park et al., 2019). Their broad neck, often accompanied by a relatively wide dome, increases the technical complexity of treatment with both microsurgical and endovascular approaches. In a meta-analysis Fiorella et al. (2017a) showed that complete occlusion rates are relatively low with both techniques, 39.8 % in the endovascular vs. 52.5 % in the microsurgical group. A comparative prospective multicenter study by Mascitelli et al. (2021) found a significantly higher occlusion rate within the microsurgical cohort (97.6 % vs. 86.5 %, p = 0.007), suggesting that traditional endovascular methods may be inferior to microsurgical clipping in the treatment of WNAs. To overcome this problem, a new endovascular device, the flow disruptor Woven EndoBridge (WEB; MicroVention, Aliso Viejo, California, USA), was introduced for the treatment of unruptured WNA in 2010 (Arthur et al., 2019). WEB is placed within the aneurysm sac to disrupt blood flow at the level of the aneurysm neck and further induce complete thrombosis of the aneurysm (Pierot, 2021). Multiple prospective studies have demonstrated that the device is a safe and effective treatment method for unruptured WNAs (Arthur et al., 2019; Pierot et al., 2018) and the device was approved for its clinical use for unruptured WNA by the US Food and Drug administration (FDA) in 2018 (Pierot, 2021). However, only a small number of ruptured aneurysms were included in the European good clinical practice (GCP) studies (Pierot et al., 2018) (8.3 %) and the WEB-IT trial (Fiorella et al., 2017b) (6.0 %). Nevertheless, neurovascular centers have used the WEB off-label in the setting of ruptured WNAs. Several recently published retrospective series and meta-analyses have reported a favorable safety profile of the WEB device in the treatment of ruptured WNAs (Cortez et al., 2021; Da et al., 2019; Essibayi et al., 2021; Harker et al., 2021; Liebig et al., 2015; van et al., 2018; Youssef et al., 2021). Both the risk of rebleeding and the procedure-related complication rate appear to be low; however, reported occlusion rates have also been relatively modest (Cortez et al., 2021; Essibayi et al., 2021; Harker et al., 2021; Liebig et al., 2015; van et al., 2018; Youssef et al., 2021), particularly when compared with outcomes following microsurgical clipping (Spetzler et al., 2015). Only a limited number of studies have directly compared WEB treatment with microsurgery (Chacón et al., 2022; Goertz et al., 2021), and, to our knowledge, no study has specifically examined microsurgical clipping versus WEB for ruptured WNAs. Based on our institutional experience and occlusion rates, supported by that of others (Mascitelli et al., 2021), we consider microsurgical clipping to offer a clear advantage over WEB in this setting. The objective of the present study was to evaluate the relative effectiveness and safety of microsurgical clipping for ruptured WNAs in comparison with published outcomes of endovascular treatment using the WEB device.

## 2 Materials and methods

### 2.1 Patient population

A retrospective study of consecutive adult patients with ruptured WNAs who underwent microsurgical clipping at our institution between 2010 and 2020 was performed. The study complied with the Declaration of Helsinki and was approved by the local ethics review committee (19/11/21). Patient's consent for treatment was obtained according to the individual institutional guidelines. Due to the retrospective analysis of the data for this study, additional informed consent for inclusion in the study was deemed unnecessary.

### 2.2 Morphological inclusion and exclusion criteria

In line with the morphological criteria deeming an aneurysm amenable for WEB treatment, WNA located at the middle cerebral artery (MCA) bifurcation, internal carotid artery terminus (ICAt), anterior communicating artery (AcomA) complex, and basilar artery (BA) apex were included in this study. Additional morphological inclusion criteria specified a neck diameter ranging from a minimum of 2.5 mm to a maximum of 8 mm, an aneurysm size between 2.8 mm and 17 mm, and a dome-to-neck ratio greater than 1 but less than 2, as per WEB indications. Aneurysms with fusiform, multilobulated, or dissecting morphology were excluded.

### 2.3 Surgical strategy

For microsurgical clipping of WNAs, a standard pterional craniotomy was performed. Intraoperative indocyanide green (ICG) angiography and micro-Doppler ultrasound were used to verify both parent and distal vessel patency. All patients underwent a postoperative computed tomography angiography (CTA) to demonstrate aneurysm occlusion and distal vessel patency, and to exclude treatment-associated infarctions or hemorrhages.

### 2.4 Outcome

#### 2.4.1 Aneurysm occlusion

The primary endpoint of this study was the complete occlusion of the aneurysm, equivalent to RROC Class I (Mascitelli et al., 2015) on postoperative CTA.

#### 2.4.2 Procedure-related morbidity and mortality

Secondary outcome measures were procedure-related morbidity and mortality. Morbidity was defined as a procedure related complication such as thrombosis, dissection, aneurysm rupture, cerebral infarction leading to a permanent neurological deficit at the time of discharge, as defined by Cortez et al. (2021). Procedure-related infarction was defined as a hypodensity present on CT or magnetic resonance imaging (MRI) between 24 and 48 h after aneurysm occlusion, not attributable to otherwise identified factors such as bleeding. Furthermore, surgical complications including surgical site infections (SSI), seizures, postoperative hemorrhage/hematoma, intraoperative aneurysm rupture, or intraoperative clipping of parent and/or distal vessel were assessed.

#### 2.4.3 Comparative cohort

We further compared our institutional occlusion rate as well as procedure-related morbidity and mortality with the data published by Cortez et al. (2021), a retrospective multicenter study within eight centers in the USA. The study included patients with ruptured intracranial aneurysms treated with WEB device in the setting of SAH. Safety outcomes were defined as periprocedural complications, including vessel perforation, thromboembolic events, and procedure-related mortality. Efficacy was assessed using the Raymond–Roy classification (I, complete occlusion; II, residual neck; III, residual aneurysm), with adequate occlusion considered as complete occlusion or residual neck.

### 2.5 Statistical analysis

IBM SPSS Statistics for Windows (Version 27.0 Armonk, NY: IBM Corp.) was used for statistical analysis. A *p*-value <0.05 was considered statistically significant. Categorical variables are presented as frequency and percentages, and continuous parameters as mean ± standard deviation (SD) or range. For group comparison between our institutional cohort and the WEB cohort, Chi Square and Fisher's exact test were used.

## 3 Results

### 3.1 Patients and aneurysms

A total of 87 patients with 87 ruptured WNAs were included. Mean age at time of ictus was 56.2 ± 13.6 years and women were more often affected than men (53:34). Clinical status according to Hunt and Hess (H&H) was grade I in 29 % (25/87), grade II in 22 % (19/87), grade III in 15 % (13/87), grade IV in 16 % (14/87), and grade V in 18 % (16/87) of patients at admission. Patient characteristics are shown in Table 1. The most common aneurysm location was the AComA complex 52 % (45/87) followed by the MCA bifurcation 44 % (38/87), ICA-t 2 % (2/87), and BA 2 % (2/87). Mean neck size was 3.8 ± 1.3 mm, mean dome size was 4.9 ± 1.8 mm, and mean dome to neck ratio was 1.3 ± 0.3 mm (Table 2).

**Table 1:** Shows patient characteristics, morphological features, and the location of the aneurysm at the time of diagnosis.

	All (n = 87)	Complete occlusion (n = 82)	Not completely occluded (n = 5)	p-value
**Age**				
Mean ± SD (range)	56.2 ± 13.6 (29–90)	57 ± 13.4 (29–90)	42 ± 9.5 (31–55)	**0.019**
**Sex**				1.00
m:f	34:53	32:50	2:3	
**Hunt&Hess**				
I-III	57 (66 %)	52 (63 %)	5 (100 %)	0.159
IV-V	30 (34 %)	30 (37 %)	–	
**Aneurysm max. diameter (mm)**				
Mean ± SD	6.2 ± 2.4	6.2 ± 2.4	5.8 ± 2.6	0.667
Median (range)	5.7 (2.8–12)	5.9 (2.8–12)	5 (2.8–9)	
**Aneurysm dome (mm)**				
Mean ± SD	4.9 ± 1.8	4.9 ± 1.8	4.5 ± 1.0	0.841
Median (range)	4.5 (2.7–11.0)	4.5 (2.7–11.0)	5.0 (3.5–5.5)	
**Aneurysm neck (mm)**				
Mean ± SD	3.8 ± 1.3	3.8 ± 1.3	3.5 ± 1.1	0.505
Median (range)	3.5 (2.5–7.7)	3.5 (2.5–7.7)	3 (2.5–4.8)	
**Dome to neck ratio**				
Mean ± SD	1.3 ± 0.3	1.3 ± 0.3	1.4 ± 0.3	0.523
Median (range)	1.3 (1.1–1.9)	1.3 (1.1–1.9)	1.4 (1.1–1.8)	
**Aneurysm location**				
AcomA	45 (52 %)	41 (50 %)	4 (80 %)	0.627
MCA bifurcation	38 (44 %)	37 (46 %)	1 (20)	
Basilar apex	2 (2 %)	2 (2 %)	–	
ICA-Terminus	2 (2 %)	2 (2 %)	–

**Table 2:** Demonstrates the complete occlusion rate and procedure-related morbidity in both treatment groups.

	WEB device n = 91	Clipping n = 87	p-value
Complete occlusion^a^			
Yes	24 (48 %)	82 (94 %)	
No	26 (52 %)	5 (6 %)	**p < 0.001**
Procedure-related complication			
Yes	3 (3 %)	2 (2 %)	
No	88 (97 %)	85 (98 %)	p = 1.00

a Follow-up angiography in the WEB cohort was available for 50/91 patients.

### 3.2 Aneurysm occlusion rate

The complete occlusion rate after microsurgical clipping was 94 % (82/87). In all patients, intraoperative ICG demonstrated complete occlusion of the aneurysm sac and vessel patency. However, postoperative imaging revealed that five aneurysms were not completely occluded: 4/5 were AcomA aneurysms and 1/5 was located at the MCA bifurcation. Retreatment was necessary in 3/5 patients; two patients were treated with coiling and one patient was treated with a WEB device.

**Fig. 1:**
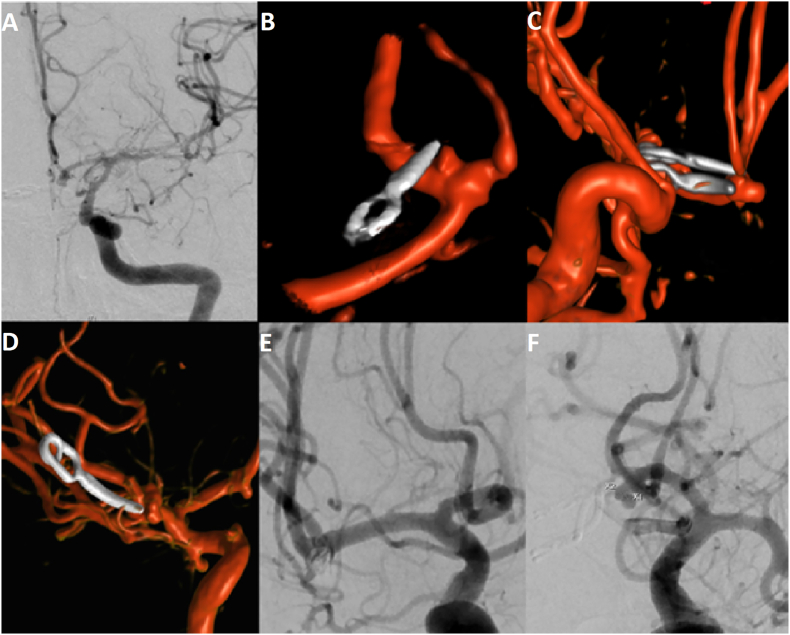
**(A**–**E)** demonstrates the postoperative imaging of the five aneurysms that were not completely occluded after microsurgical clipping. **(A)** and **(B)** display small neck-remnants after clipping of an AcomA aneurysm with one clip. **(C)** postoperative angiography after the rebleeding demonstrates aneurysm remnant located at the AcomA complex. **(D)** shows an aneurysm remnant located at the media bifurcation and **(E)** the final outcome after retreatment with coiling. **(F)** demonstrates incomplete occlusion (RROC II) of an AcomA aneurysm after microsurgical clipping.

The first patient presented with a ruptured wide-necked AcomA aneurysm with a diameter of 8 mm and a dome-to-neck ratio of 1.1. Clinical status at admission was H&H grade II. The aneurysm was treated with a single straight clip. Postoperatively, the patient developed an increase in transcranial Doppler (TCD) flow velocities, severe vasospasm was diagnosed on CTA, and the patient had to undergo percutaneous transluminal angioplasty (PTA) on day five after ictus. Besides severe vasospasm of the left anterior cerebral artery (ACA) and MCA, the angiography showed a small neck-remnant of 2 mm (Fig. 1A). In interdisciplinary consensus with interventional neuroradiology, due to the small size no further treatment of the neck remnant was performed.

The second patient suffered an aneurysmal SAH H&H grade II caused by a ruptured AcomA aneurysm with a diameter of 2.8 mm and dome-to-neck ratio of 1.4. The patient underwent microsurgical clipping and the aneurysm was occluded with a single straight clip. Postoperative CTA demonstrated residual filling of the aneurysm and the patient underwent a follow-up angiography for further evaluation, which confirmed a neck-remnant of 3.0 × 1.6 mm (Fig. 1B). However, after interdisciplinary discussion, retreatment was not indicated for this small remnant.

The third patient was diagnosed with an aneurysmal SAH on CT/CTA. Clinical status at time of admission was H&H grade III. Digital subtraction angiography (DSA) demonstrated a ruptured AcomA aneurysm with a diameter of 4 mm and a dome-to-neck ratio of 1.1. The aneurysm was occluded with two straight clips. The surgery and postoperative course were uneventful. However, two weeks after microsurgical clipping, an acute clinical deterioration occurred with anisocoria and a mydriatic pupil on the left side. A CT and CTA were performed immediately and demonstrated a left sided subarachnoid rebleeding. The patient underwent an emergency DSA and a ruptured aneurysm remnant was diagnosed as the source of the rebleeding (Fig. 1C). Given the broad base of the remnant, the neuroradiology team deemed coiling unsuitable. Re-clipping was excluded due to cerebral swelling, the patient's vulnerable condition during the vasospasm phase, and associated surgical risks. Furthermore, considering the potential need for additional cerebrospinal fluid (CSF) diversion, we sought to avoid treatments requiring dual antiplatelet therapy (DAPT). Following interdisciplinary discussion, WEB device was selected as the most appropriate treatment option for this patient. The patient recovered well and was discharged without any neurological deficits.

The fourth patient presented with a SAH H&H grade II. The source of the bleed was a ruptured MCA aneurysm with a diameter of 9 mm and neck-to-dome ratio of 1.8. Postoperative CTA suggested a neck remnant and a DSA was performed for further diagnosis. DSA confirmed a remnant, equivalent to RROC grade 2 (Fig. 1D). To avoid a second surgery during the vasospasm phase, and given the configuration of the remnant, adjunctive coiling was performed (Fig. 1E).

The fifth patient presented with an aneurysmal SAH H&H grade I caused by a ruptured wide-necked AcomA aneurysm with a diameter of 5 mm and a dome-to-neck ratio of 1.4. Microsurgical clipping was performed, and the aneurysm was occluded with two straight clips. However, postoperative DSA showed incomplete occlusion RROC grade 2 (Fig. 1F). Given the vasospasm phase, re-clipping was again deemed unsuitable, and endovascular coiling was selected as the treatment of choice owing to the small neck of the remnant.

### 3.3 Surgical adverse events, morbidity, and mortality

The procedure-related morbidity at the time of discharge was 2.3 % (2/87) in our cohort. The first patient presented with a SAH H&H grade IV. DSA demonstrated a ruptured MCA bifurcation aneurysm with a diameter of 5 mm and a dome-to-neck ratio 1.8. The aneurysm was clipped with a single straight clip. Although intraoperative micro-Doppler ultrasound and ICG angiography demonstrated vessel patency of both M2 branches after clip application, occlusion of a M2 branch distal to the aneurysm clip was diagnosed on postoperative CTA, most likely due to a thromboembolic event. This further caused an infarction of the right MCA territory and led to a left sided severe hemiparesis (M 1/5) at the time of discharge. However, the patient recovered well and was only left with a residual left sided impairment of the fine motor skills at the one-year follow-up.

The second patient who was left with a procedure related neurological deficit at discharge was a 60-year-old patient who suffered from an SAH H&H grade II caused by a ruptured AcomA aneurysm with a diameter of 11 mm and a dome-to-neck ratio of 1.3. The postoperative CT demonstrated an infarction of the left anterior cerebral artery (ACA) which led to a severe right sided paresis (M 1/5) at the time of discharge into a neurological rehabilitation centre. Unfortunately, further clinical follow-up was not available.

Other surgical adverse events included SSI, hygroma, and cerebral spinal fluid (CSF) fistula in one patient (1 %, (1/87)), respectively. Furthermore, 5 % (4/87) of aneurysms ruptured intraoperatively, however, this did not lead to new neurological deficits in any patient. Mean follow-up in our cohort was 22.7 (±29.1) months. Neurological outcome according to the mRS was ≤3 in more than 60 % (53/87) of all patients at time of last follow-up. Patients who presented with a higher H&H grade at time of admission also had a higher mRS i.e. a worse clinical outcome at the time of the last follow-up (p < 0.001).

#### 3.3.1 Comparative cohort

The comparative cohort by Cortez et al. (2021) evaluated patients with ruptured intracranial aneurysms treated with WEB. A total of 91 patients with 94 aneurysms were included, with a female predominance (68.1 %) and a mean age of 57.3 ± 15.4 years. The most frequent aneurysm locations were the anterior communicating artery (44.6 %, 42/94), middle cerebral artery (17 %, 16/94), and basilar artery (16 %, 15/94). The mean dome size was 5.7 ± 1.9 mm (range 2.4–11), while the maximum aneurysm diameter averaged 6.9 ± 2.6 mm (range 3.3–16). The mean dome-to-neck ratio was 1.6 ± 0.5, and the mean neck size was 3.7 ± 1.5 mm. Of the aneurysms, 78 (82.9 %) were classified as wide-necked, defined as having either a neck ≥4 mm or a dome-to-neck ratio ≤2.0.

#### 3.3.2 Procedure-related morbidity and mortality

Treatment was successful in 97.8 % (89/91) of patients. Parent vessel flow limitation occurred in 4.5 % (4/91); one case was converted to clipping, while three required adjunctive stenting. Intraoperative thrombus was seen in 6.7 % (6/91), resulting in symptomatic ischemia in three patients. Aneurysm rupture occurred intraoperatively in three cases (3.2 %). Overall, symptomatic procedure-related complications occurred in 4.5 % (4/91), mainly ischemic (75 %). Procedure-related morbidity at discharge was 3.3 % (3/91). Mortality at discharge was 13.9 % (12/91), all attributable to the initial SAH and not to procedural complications.

#### 3.3.3 Occlusion rate

Angiographic follow-up was available for 50 patients, with a median follow-up of 3.4 months (IQR 2–6). Complete aneurysm occlusion was achieved in 48.0 % (24/50) of patients, while 32.0 % (16/50) showed a residual neck and 20 % (10/50) a residual aneurysm. Retreatment of the target aneurysm was required in 8.0 % (4/50). No cases of delayed mortality or re-rupture were reported.

#### 3.3.4 Clip or WEB?

Table 2 summarizes occlusion rates and procedure-related sequelae in both groups. Procedure-related morbidity was 3.3 % (3/91) in the endovascular cohort and 2.3 % (2/87) in the microsurgical cohort. Statistical analysis demonstrated that both techniques were associated with a comparably low risk of procedure-related complications. The complete occlusion rate was 94 % (82/87) in our microsurgical cohort, which was significantly higher than the 48 % (24/50) reported in the multicenter WEB cohort (p < 0.001). Mortality related to subarachnoid hemorrhage was approximately 13 % in both groups (12/91 in the endovascular and 11/87 in the microsurgical cohort), whereas procedure-related mortality was 0 % in both cohorts.

## 4 Discussion

The introduction of WEB in 2010 has revolutionized the endovascular treatment of ruptured WNAs. Due to their unfavorable configuration, the treatment of WNAs poses a treatment challenge with traditional endovascular methods (Fiorella et al., 2017a). Over the past decades, several techniques and additional devices, such as stent and balloon assisted coiling or flow-diversion, have been developed to achieve complete aneurysm occlusion in those cases. However, the use of these devices leads to a higher complexity of the procedure and is consequently associated with a higher risk for complications. Additionally, deployment of specific devices within the parent artery is associated with an elevated risk of arterial thrombosis, thereby warranting the use of periprocedural DAPT. This, on the other hand, is associated with a higher risk of early rebleeding in the postoperative period (van Rooij et al., 2017). Thus, one of the major advantages of WEB is that it is deployed within the aneurysm sac and anticoagulation can be limited to periprocedural heparinization (van Rooij et al., 2017). This consideration is particularly relevant in the context of SAH, where subsequent invasive interventions, including external ventricular drain (EVD) placement, lumbar drainage, or permanent CSF diversion, may be required.

Several retrospective series and meta-analyses on the use of WEB in ruptured WNAs reported a low rate of procedure-related complications and a good efficacy in preventing rebleeding (Cortez et al., 2021; Harker et al., 2021; Liebig et al., 2015; van et al., 2017). A recent meta-analysis including 285 patients from 16 studies by Essibayi et al. (2021) reported a procedure-related complication rate of 3.2 % and the rebleeding rate was found to be 1.1 %. The interim results of the prospective European multicenter trial - CLinical Assessment of WEB device in Ruptured aneurysms (CLARYS) have also demonstrated a low periprocedural risk (3.3 %) and a low rate of rebleeding (Spelle et al., 2021). Those results are similar to the findings in our microsurgical cohort and the study by Cortez et al. (2021) with a procedure-related morbidity of 2.3 % (2/87) and 3.3 % (3/91), respectively.

The CLARYS study is currently the only prospective study that has evaluated the WEB for the treatment of ruptured aneurysms with a complete occlusion rate of 41 % (19/46). Within the limited follow-up time of 12 months a retreatment rate of 13 % has been reported (Spelle et al., 2022). A higher retreatment rate for ruptured than for unruptured aneurysms has also been demonstrated in two large multicenter studies (Caroff et al., 2022; Srinivasan et al., 2022). Furthermore, an increased rebleeding rate (7.7 % vs. 0.6) for initially ruptured aneurysms treated with the WEB has been shown (Srinivasan et al., 2022). So far, those numbers are limited to a short follow-up time and the rate of remnants and recurrences as well as the risk of rebleeding after WEB treatment of ruptured aneurysms may increase over time. Prospective studies with a long-term follow-up after microsurgical clipping of ruptured aneurysms on the other hand have demonstrated low rebleeding and a low retreatment rates (Spetzler et al., 2015; Molyneux et al., 2015). This further emphasizes that microsurgical clipping is an evidence-based safe and effective treatment option for ruptured WNA. Another major advantage of microsurgical aneurysm clipping, especially in patients with ruptured aneurysms, is the immediate and complete aneurysm occlusion, which remains one of the most important treatment goals; especially in ruptured aneurysms, not only to prevent a re-rupture and its fatal consequences, but also to avoid further aneurysm treatment and interventional follow-up imaging.

Although it has been postulated that neck and aneurysm remnants after WEB treatment may occlude over a longer time period, current data for unruptured lesions rather indicates the opposite. At 3 years, complete occlusion in 50.8 %, neck remnant in 32.8 %, and aneurysm remnant in 16.4 % has been reported in the cumulative population of the WEBCAST and WEBCAST 2 study (Pierot et al., 2021). After 5-years, complete aneurysms occlusion was seen in 51.6 %, neck remnants in 26.3 %, and an aneurysm remnant in 22.1 % (Pierot et al., 2022). The retreatment rate was 11.4 % and 11.6 % after 3 and 5 years of follow-up, respectively (Pierot et al., 2021, 2022). Since the retreatment rates in ruptured aneurysms treated with WEB have been reported to be higher over a shorter follow-up period, the available data on unruptured aneurysms suggests that the number of aneurysm remnants in ruptured lesions may increase even more over a longer follow-up period.

Although a good safety profile of the WEB device has been demonstrated, the previously reported complete occlusion rates of approximately 40–60 % seem quite low compared to other treatment methods, especially compared to microsurgical clipping (Spetzler et al., 2015, 2019; Molyneux et al., 2015). At this point it is also important to mention that most studies evaluating WEB do not report a complete but an “adequate” occlusion rate which is a controversially discussed term within the scientific community. Essibayi et al. (2021) for example have reported an adequate occlusion rate of 87.3 % which is similar to the complete occlusion rates with microsurgical clipping. However, the term “adequate” occlusion includes aneurysms with a WEB occlusion scale (WOS) A, B, and C, where WOS C describes a neck remnant equivalent to RROC II (Arthur et al., 2019). Thus, the subcategory “adequate” also includes incompletely occluded aneurysms (RROC II) and may distort the real treatment outcome. However, the complete occlusion rate in our microsurgical group is still significantly higher than the adequate occlusion rate reported with WEB in the meta-analysis by Essibayi et al. (2021).

In summary, microsurgical clipping is an immediate, well-established, safe, and effective treatment option for ruptured aneurysms whereas WEB needs further evaluation and current data has to be interpreted carefully due to the lack of a long-term follow-up. However, only a randomized trial comparing the two methods regarding their efficacy and safety will be able to answer the question of superiority in the treatment of ruptured WNAs.

## 5 Conclusion

The study demonstrates that microsurgical clipping is a safe and effective treatment method for ruptured WNAs and that it is superior to the endovascular treatment with the WEB device in terms of complete occlusion rates. Our study further shows that the WEB device is not superior to surgical treatment regarding procedure-related complications.

### 5.1 Limitations

One limitation to the study is its retrospective character. Furthermore, there may exist a selection bias. Patients eligible for microsurgery as well as for endovascular treatment were selected according to numerous patient and aneurysmal criteria and may have been chosen in favour to one or the other method. A propensity matched-pair analysis might have been able to correct for heterogeneity between the endovascular and microsurgical groups, but it was beyond this study's design. Clearly, a prospective evaluation of both modalities would be the ideal setting. Another limitation is that CTA was used as an imaging modality after microsurgical clipping. We do acknowledge the fact that that postoperative angiography is superior to CTA for the demonstration of complete aneurysm occlusion. Additionally, we did not evaluate the clinical outcome extensively, as the main focus of this study was a morphological/imaging one. To which extent complete occlusion might influence long-term clinical outcome is uncertain, and our study was not powered or designed to answer this question. Furthermore, our results might not be representative for general vascular neurosurgery since our institution is a tertiary referral center with a large number of microsurgical aneurysm clippings, which consequently may lead to a more favorable outcome and distort morbidity and the occlusion rate in our cohort.

## Author contributions

All authors contributed to the study conception and design. Material preparation, data collection and analysis were performed by Beate Kranawetter. The first draft of the manuscript was written by Beate Kranawetter and all authors commented on previous versions of the manuscript. All authors read and approved the final manuscript.

## Ethical approval

The study complied with the Declaration of Helsinki and was approved by the local ethics review committee (19/11/21).

## Declaration of competing interest

The authors declare that they have no known competing financial interests or personal relationships that could have appeared to influence the work reported in this paper.
